# Bayesian Selection Policies for Human-in-the-Loop Anomaly Detectors with Applications in Test Security

**DOI:** 10.1017/psy.2025.10056

**Published:** 2025-12-10

**Authors:** Michael Fauss, Xiang Liu, Chen Li, Ikkyu Choi, H. Vincent Poor

**Affiliations:** 1 ETS Research Institute, USA; 2 Department of Electrical and Computer Engineering, Princeton University, USA

**Keywords:** anomaly detection, human in the loop, test security, variational Bayes

## Abstract

This article investigates the problem of automatically flagging test takers who exhibit atypical responses or behaviors for further review by human experts. The objective is to develop a selection policy that maximizes the expected number of test takers correctly identified as warranting additional scrutiny while maintaining a manageable volume of reviews per test administration. The selection procedure should learn from the outcomes of the expert reviews. Since typically only a fraction of test takers are reviewed, this leads to a semi-supervised learning problem. The latter is formalized in a Bayesian setting, and the corresponding optimal selection policy is derived. Since calculating the policy and the underlying posterior distributions is computationally infeasible, a variational approximation and three heuristic selection policies are proposed. These policies are informed by properties of the optimal policy and correspond to different exploration/exploitation trade-offs. The performance of the approximate policies is assessed via numerical experiments using both synthetic and real-world data and is compared with procedures based on off-the-shelf algorithms as well as theoretical performance bounds.

## 1 Introduction

A common task when administering and evaluating tests is to identify behaviors or responses that are atypical and may require special attention to ensure the integrity of the test (Bulut et al., 2024; Cizek & Wollack, 2016; He et al., 2022; Kingston & Clark, 2014; Wongvorachan, 2023). For example, in writing tests, some responses might need closer inspection to establish if they were plagiarized (Foltýnek et al., 2019; Gomaa & Fahmy, 2013) or, more recently, AI-generated (Jiang et al., 2024; Yan et al., 2023). In language tests, test takers might be reading off scripts or repeating after a hidden *souffleur* instead of speaking freely (Evanini & Wang, 2014; Wang et al., 2016, 2019). Similar tasks also occur outside a fraud detection context. For example, one might want to identify test takers that did not properly engage with the items (Booth et al., 2023; Cocea & Weibelzahl, 2010), that should have been provided with certain accommodations (Kettler, 2012; Sireci et al., 2005), or are not using the provided equipment as intended (Taylor et al., 1998).

In many such scenarios, the respective test takers or responses are first *flagged* by automated systems. For example, given the large amount of potential source material, initial plagiarism checks are almost exclusively done via automated systems (Jiffriya et al., 2021). However, especially in high-stakes testing, relying on automated flags alone is risky because of inevitable false positives. Therefore, once a test taker has been flagged by an automated system, their case is typically reviewed in more detail before a final decision is made. The subject of this article is the design of an automated system that flags test takers for review and learns from their outcomes. Since in a testing context such reviews are typically carried out by one or more human experts, we refer to this scenario as *human-in-the-loop* anomaly detection. However, the proposed procedure is, in principle, agnostic to how the reviews are conducted.

Before going into more detail, it is useful to fix some terms. In what follows, we refer to the group of test takers that we seek to identify as *critical group*. All test takers that are not members of the critical group are referred to as the *reference group*. Moreover, we refer to test takers that are identified by the automated system as *flagged* or *selected* for review. If the review outcome is positive, that is, the test taker is indeed found to be a member of the critical group, we refer to the corresponding flag as a *detection* or *true positive*. If the review outcome is negative, that is, the test taker is found *not* to be a member of the critical group, we refer to the flag as a *false alarm* or *false positive*. The rule by which test takers are selected for review is referred to as a *flagging* or *selection policy*. Finally, depending on the context, we will say that a *test taker* is selected for review or that a *response* is selected for review. For the purpose of this article, this difference is insubstantial.

We assume that a test is administered periodically over time. The flagging procedure we aim to design is assumed to work as follows: for each test taker in the current administration, the automated system is provided with a set of features that were extracted from their response and/or behavior. The system processes these features and outputs a binary flagging decision. The goal is to find a policy that maximizes the number of test takers correctly identified as needing special attention (true positives), while keeping the number of required reviews per test administration at a manageable level. The latter is important since, depending on what it entails, an expert review can require a significant amount of resources. The features based on which the flagging decision is made are not subject to this optimization, but are assumed to be given. We refer to this problem as the *flagging problem*. It will be defined more formally in Section 2.

Although the methods proposed in this article potentially cover a wide range of applications, we envision them to be most useful in settings where the outcomes of multiple detectors or classifiers need to be fused (Varshney, 2012). For example, returning to the task of plagiarism detection, instead of relying on a single checker, multiple checkers might be run on a submitted response, each of them returning a value that quantifies how likely the response was plagiarized. A decision whether or not to flag the response then needs to be made by fusing the results of the individual checkers into a single, binary outcome.

In order to position this article in the context of existing research, it is useful to discuss some of the characteristics and requirements of the system we seek to design:

- We do not assume the existence of a (large) training data set. This assumption is somewhat pessimistic, but often realistic. For example, when new item types are introduced or new ways of cheating emerge, little to no data will be available to train detectors.
- We assume that a test taker’s group membership can be established via an expert review. However, typically only a fraction of test takers will undergo a review, thus rendering the flagging problem a *semi-supervised* learning problem (Van Engelen & Hoos, 2020).
- In contrast to many semi-supervised learning problems, we do not assume the subset of labeled data points to be given in advance. Instead, the selection policy itself determines which data points are reviewed and labeled; in a sense, the system can and must select its own training data—a concept commonly found in *active learning* (Felder & Brent, 2009).
- We consider data fusion the main use case of the system we seek to design. Hence, it should be able to handle “reasonably high-dimensional” feature spaces, say, on the order of tens or hundreds of features, but is not necessarily expected to process very large feature vectors commonly used in supervised machine learning (Caruana et al., 2008).
- We seek to avoid strong assumptions on the number of test takers or the number of reviews conducted. That is, the system should neither require a minimum sample size, nor should compute or memory requirements limit its application to small-scale tests.
- In order to learn and track the feature distributions of interest, the system should be adaptive and operate in a continuous feedback loop. That is, the outcomes of expert reviews are fed back into the system, which then uses this feedback to update its parameters and in turn improve the accuracy of its selection policy.
- Especially in a high-stakes testing context, it is crucial that important metrics of the automated selection system, such as its false positive and false negative rate, can be evaluated or at least estimated. In the scenario considered here, this is not straightforward since only selected test takers (positives) are reviewed. Hence, metrics, such as the false negative rate, cannot be evaluated empirically. This limits the applicability of commonly used classifiers whose outputs can be interpreted as probabilities, but are not based on probabilistic models (Bielza & Larrañaga, 2020).

In light of these requirements, we propose a Bayesian approach to the flagging problem. In particular, as will be shown in the course of the article, a Bayesian approach allows for a unified treatment of labeled and unlabeled data points, and in turn enables a seamless implementation of a feedback loop. Moreover, prior distributions make it possible to incorporate qualitative prior knowledge in cases where little or no training data is available. As more samples are collected, this prior knowledge is gradually overwritten by evidence learned from the data. A disadvantage of the Bayesian approach is that both the inference step, that is, the update of the posterior distribution, and the optimal selection policy are too complex to be implemented in practice. We address this problem by, first, using a variational approximation of the posterior distribution, and, second, proposing three heuristic selection policies corresponding to different exploration/exploitation trade-offs.

Naturally, Bayesian approaches have been used in the context of test integrity before (see, for example, Lu et al., 2023; Marianti et al., 2014; Van der Linden & Guo, 2008; van der Linden & Lewis, 2015; Zhang et al., 2022 to name just a few). Moreover, some Bayesian methods have been proposed for general semi-supervised learning problems (Adams & Ghahramani, 2009; Bruce, 2001; Rottmann et al., 2018). However, the specific scenario considered in this article, learning sequentially from self-selected subsets of batched data, is non-standard and non-trivial. To the best of our knowledge, it has not been investigated in detail before, and methods in the literature did not meet all of the requirements listed above.

Finally, we would like to highlight that, although they were motivated by a test integrity scenario, both the problem formulation and the inference and flagging procedures presented in this article are generic and extend beyond this specific application. In principle, the proposed methods apply whenever noisy measurements are used to select individuals or objects for expert inspection. This is the case, for example, in industrial quality control, where sensor data is used to monitor and flag defective products for manual inspection (Mitra, 2016), in condition-based maintenance, where predictive models are used to determine when machinery needs servicing based on wear and usage data (Prajapati et al., 2012), and in cybersecurity, where anomalous network activity might be flagged for further review by security experts (Ahmed et al., 2016).

The remainder of the article is organized as follows: In Section 2, we introduce our notation and discuss the problem formulation and the underlying assumptions in a more formal manner. The corresponding optimal selection policy is presented and discussed in Section 3. In Section 4, a variational approximation of the true posterior distribution is presented and three heuristic selection policies are proposed that are informed by properties of the optimal policy and correspond to different exploration/exploitation trade-offs. Two theoretical performance bounds are stated in Section 5. In Section 6, numerical examples are given that demonstrate the proposed procedure and compare its performance to that of off-the-shelf algorithms. Section 7 concludes the article and provides a brief outlook on open questions and possible extensions.

## 2 Problem formulation and assumptions

In this section, we introduce our notation, state our assumptions, discuss the underlying information flow, and give a formal definition of the flagging problem.

### 2.1 Notation

Random variables are denoted by uppercase letters, *X*, and their realizations by the corresponding lowercase letters, *x*. Analogously, uppercase letters, *P*, denote probability distributions and the corresponding lowercase letters, *p*, denote probability density functions (PDFs). Occasionally, subscripts are used to indicate a distribution or density of a certain random variable, say, 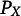 and 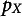 . We use 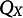 and 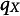 to indicate that the corresponding distribution or density is closely related but not identical to 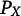 or 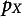 , respectively. For example, 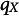 might be an approximation or unnormalized version of 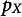 . The expected value of a random variable is written as 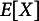 . Again, a subscript is used to explicitly indicate the random variable, say, 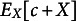 . Equality in distribution is denoted by 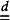 . Unless stated otherwise, collections of variables, such as vectors and matrices, are indicated by bold font. We write 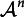 to denote the *n*-fold Cartesian product of a set 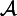 with itself. The *n*-dimensional probability simplex is denoted by 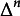 . The set of Boolean vectors of length *N* whose elements sum to *K* is denoted by 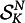 . The set of all positive semi-definite matrices of size *M* is denoted by 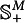 . We further define the elliptope of correlation matrices as 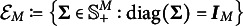, and the projection of a matrix 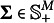 on 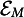 as 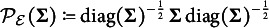. Here, 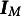 denotes the identity matrix of size *M* and 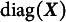 denotes the diagonal matrix with the same main diagonal elements as 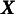 . Additional symbols and notations will be defined when they occur in the text.

We assume that a test is administered periodically at time instances 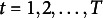 . Each administration is assumed to have 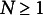 test takers.^1^ An unknown share of test takers is assumed to belong to the critical group whose members we seek to flag. In order to formalize the flagging problem, we introduce the following random variables:

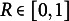
 denotes the rate of critical group members, that is, the probability that a randomly selected test taker is a member of the critical group. *R* is assumed to be a latent variable.

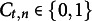
 indicates whether the *n*th test taker in the *t*th administration is a member of the critical group ( 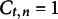 ) or not ( 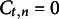 ). 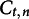 is assumed to be a latent variable.

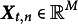
 , 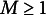 , denotes the vector of features associated with the *n*th test taker in the *t*th administration. 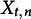 is assumed to be observable and provided by a given mechanism.

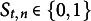
 indicates whether the *n*th test taker in the *t*th administration was selected for review ( 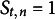 ) or not ( 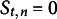 ). 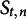 is set according to the chosen selection policy.

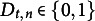
 denotes whether the *n*th test taker of the *t*th administration was detected as being a member of the critical group ( 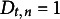 ) or not ( 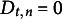 ). 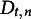 is the outcome of an expert review and can be observed.

For the sake of a more compact notation, variables corresponding to the same administration are also written as column vectors:

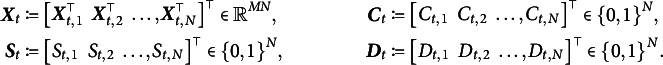

Analogously, horizontal stacks of vectors of the first *t* administrations are written as

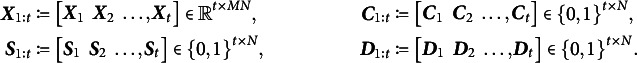



### 2.2 Information flow

Before going into more details of the model underlying the random variables defined in the previous section, it is useful to highlight how the available information evolves over time. Before the administration at time instant 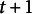 , the available data consist of the features of all previous test takers, 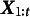 , the subset of test takers selected for review, 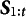 , and the outcomes of these reviews, 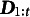 . We denote the 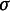 -algebra of events generated by these random variables by
(1)

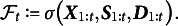

After the administration at time instant 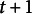 is completed and all responses are processed, the extracted features, 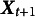 , become available. We denote this refined 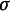 -algebra by
(2)

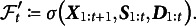

Note that the decision about which test takers to select for review should take 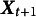 into account, hence, it can use all information available in 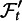 , not just 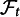 . Finally, the selected responses, 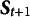 , and the outcomes of the corresponding reviews, 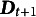 , are observed. This refines the 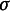 -algebra to 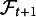 and completes the cycle. Note that 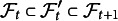 for all 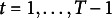 .

### 2.3 Assumptions

Throughout the article, we make the following assumptions:

1. All group membership indicators, 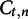 , are independent and identically distributed Bernoulli random variables with success probability *R* so that 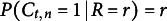 .
2. All feature vectors, 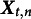 , are independent and identically distributed conditioned on the group membership indicator 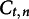 . That is, there exist two random vectors, 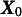 and 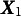 , such that 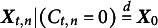 and 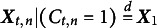 for all *t* and *n*. The distributions of 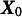 and 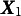 are denoted by 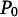 and 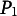 , respectively.

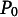
 and 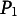 are assumed to be members of two, not necessarily identical, parametric families of distributions. That is, there exist two (vector) parameters in suitably defined spaces, 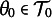 and 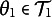 , such that
(3)

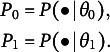

4. The selection process is modeled as follows: After each administration, 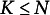 test takers are selected for review. The corresponding selection policy is a function 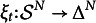 that assigns a probability to every possible selection vector. Each 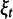 is assumed to be 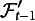 -measurable, that is, the selection policy can depend only on events whose occurrence can be established based on knowledge of 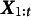 , 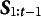 , and 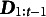 . Note that this implies that 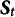 and 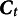 are conditionally independent since
(4)

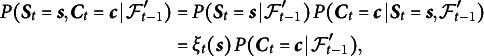

where the second equality holds since 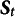 can only provide information that is already contained in 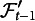 .
5. The review process is modeled as follows: All flagged test takers are reviewed and the review resolves any ambiguity about the test taker’s group membership. Test takers that did not get flagged are automatically assumed to be members of the reference group. Unflagged members of the critical group remain undetected. This means
(5)

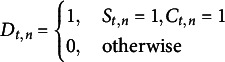

or, using Hadamard’s element-wise vector product notation,
(6)

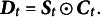

Assumptions 1 and 2 are made to simplify the mathematical analysis and to keep the model general. Possible relaxations, such as allowing dependencies between test takers (and possibly re-takers), or considering sub-populations with different feature distributions or rates of critical group members, will significantly complicate the analysis and likely depend on what behavior or phenomenon underpins the definition of the critical group. A brief outlook on possible research avenues in this direction will be given in Section 7.

Assumption 3 enables us to formulate the flagging problem in a standard Bayesian setting. An extension to a non-parametric formulation is beyond the scope of the article.

Assumption 4 is common in sequential decision making and ensures that the policy does not use information that is not available at the time the selection decision needs to be made.

Assumption 5 is likely the most controversial, and it will not always hold in practice. However, at least in the context of test security, various standards and criteria have been established according to which an expert or a panel of experts can evaluate the evidence and arrive at a well-justified decision whether or not a certain response or behavior constitutes cheating. Alternatively, one can define the critical group as those test takers who an expert considers “sufficiently curious” to be reviewed in detail, irrespective of the outcome of the review. This criterion is less sharp, but can be more applicable in practice. In general, we assume that some, potentially expensive and time-consuming, process exists that assigns “true positive” and “false positive” labels to the selected cases. The proposed procedure is agnostic to the exact meaning of these labels and the process used to assign them.

### 2.4 Problem formulation

As mentioned before, we adopt a Bayesian perspective in this article. That is, we assume that unknown quantities are themselves random variables, and that the joint distribution of all random variables, latent and observable, is known. We denote this joint distribution by *P*, so that
(7)

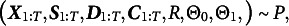

where 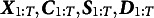 , and *R* are defined in Section 2.1, and 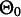 and 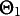 denote the parameters of the conditional feature distributions in (3).

Our aim is to design a selection policy, 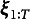 , that maximizes the expected number of detected members of the critical group, while limiting the number of expert reviews per test administration to at most 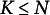 . This leads to the following optimization problem:
(8)

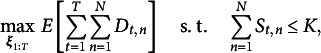

where the constraint needs to hold almost surely for all 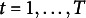 .

Clearly, (8) is by no means the only way of formalizing the flagging problem. For example, one could relax the constraint to hold in expectation, constrain the false or true positive rate instead of the absolute number of reviews, or, in a more traditional Bayesian formulation, define costs of reviews, detections, false alarms, and so on. Nevertheless, we believe that the problem formulation in (8) strikes a good balance between transparency, tractability, and applicability. In particular, the question of how to set *K*, that is, the number of reviews one is willing to afford, is well-defined and can be understood and discussed without deeper technical knowledge. By contrast, choosing Bayesian costs is typically less straightforward and can lead to confusion about what these cost should and should not reflect. Moreover, we believe that the optimal and approximate selection policies presented in this article provide a blueprint that can be adapted to variations of the problem in (8) in a relatively straightforward manner.

## 3 Optimal selection policy

This section states and discusses the selection policy that solves the problem in (8). While typically too complex for practical use, it offers conceptual insights and can guide the design of approximate or heuristic policies.

Theorem 1. Let 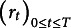 and 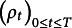 with 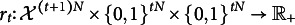 and 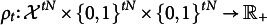 be two sequences of functions that are defined recursively via
(9)

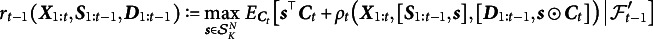

and
(10)

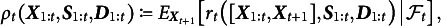

with recursion base 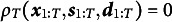 . Let 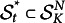 be the set of selection vectors that attain the maximum on the right-hand side of (9). Every selection policy whose probability is concentrated on the sets 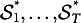 , that is,
(11)

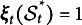

for all 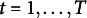 is optimal in the sense of (8).

Theorem 1 is proven in Appendix A. The functions 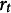 and 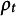 represent two types of expected rewards. Specifically, 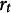 denotes the expected number of critical group members that will be flagged in the remaining 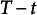 test administrations, given the data from all previous administrations and the feature vectors of the next one. The function 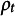 is then obtained by marginalizing 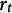 over the feature vectors of the next administration with respect to their current posterior predictive distribution. In what follows, we will occasionally refer to 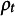 as a *look-ahead* step or function.

The optimal selection policy will be discussed in more detail shortly. Before doing so, it is instructive to investigate how the underlying probability distributions evolve as more data become available and how the conditional expectations in (9) and (10) can be calculated.

### 3.1 Posterior update

As more tests are administered and more reviews are conducted, more information is gathered that needs to be incorporated into the underlying Bayesian model. In this section, we detail the corresponding updates and provide expressions for the respective distributions. Of particular interest is the joint posterior distribution of 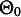 , 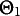 , and *R* since the latter are assumed to persist between test administrations. That is, 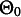 , 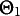 , and *R* can be learned from the data, while the uncertainty about the group indicators, 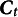 , will not be resolved completely in general.

Assume that the administration at time instant 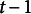 was completed, and let the corresponding conditional PDF of the persistent random variables be denoted by 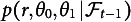 . Now, consider the joint distribution of 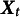 and 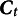 conditioned on the model parameters:
(12)

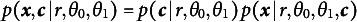



(13)

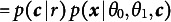



(14)

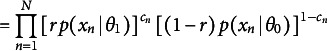



(15)

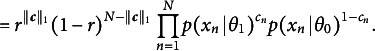

Since conditioning on the model parameters renders the distribution independent of the specific administration, we dropped the time index in the notation. By marginalizing out the unknown group membership, we obtain the feature distribution for a given set of model parameters:
(16)

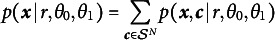



(17)

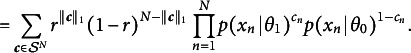

Averaging over the unknown model parameters yields the posterior predictive distribution of the features at time instant *t*:
(18)

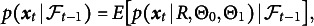

where the expected value is taken with respect to the current model posterior, 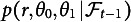 . Note that the posterior predictive in (18) is needed for the look-ahead step in (10).

Now, assume that the test administration at time instant *t* was completed and that 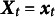 was observed. In order to determine the optimal selection policy in (9), we require the posterior distribution of the group memberships, 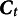 , conditioned on all previous observations. According to Bayes’ rule, this distribution is given by
(19)

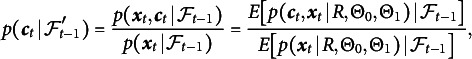

where, again, all expected values are taken with respect to 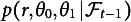 .

Given (19), the optimal selection vector can, in principle, be determined by finding the maximum in (9), and the corresponding test takers can be reviewed. In our model, this corresponds to observing 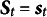 and 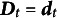 . This new information leads to the model update
(20)

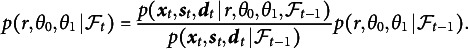

The likelihood in the numerator of (20) is given by
(21)

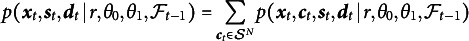



(22)

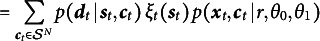



(23)

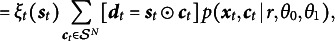

where we used Iverson bracket notation as a shorthand for the indicator function, meaning that the summands on the right-hand side of (23) are zero unless 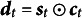 holds. Now, define
(24)

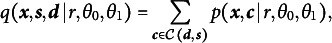

where 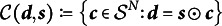. Note that 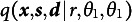 in (24) and 
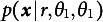
 in (17) are closely related. While the latter is obtained by summing over *all* possible group memberships, the former is obtained by summing only over those that are compatible with the observed review outcomes. This implies that (24) needs to be normalized to be a valid PDF. The final posterior update is then given by
(25)

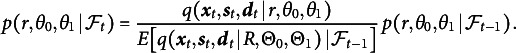

**Figure 1:**
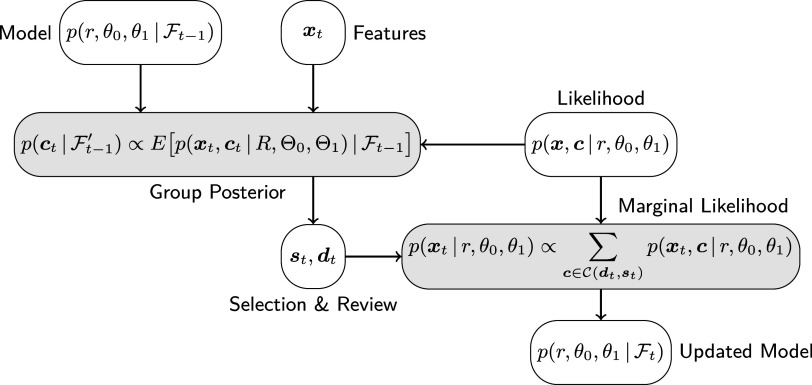
Graphical illustration of information flow and posterior update of the Bayesian model.

The factor 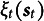 in (23) is 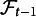 -measurable and cancels out in the normalization. The update steps now repeat with 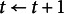 (see Figure 1 for a graphical illustration of the process).

### 3.2 Discussion

In this section, we briefly discuss the general structure and some properties of the optimal selection policy stated in Theorem 1. This discussion will also provide qualitative insights that can inform the design of approximate procedures.

By inspection, the optimal selection policy in (9) maximizes the expected value of a sum of two terms, 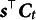 and 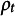 . The first term corresponds to the number of critical group members detected in the current administration; the second term corresponds to the expected number of critical group members detected in all future administrations. This trade-off between immediate and future rewards is typical for sequential decision-making procedures. However, there are a few aspects that are non-standard:

- In general, the optimal policy at time *t* depends on all data observed so far and requires averaging over all future data. This results in extremely high complexity, even for small *T* and *N*, making strictly optimal policies virtually impossible to compute in practice.
- In line with the information evolving in two steps, compare Section 2.2, the calculation of the expected reward in Theorem 1 is split into two parts. First, in (9), the review outcomes are predicted based on the observed features, 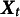 . Then, in (10), the features themselves are predicted based on the data from previous administrations. These two expectations correspond to two different sources of uncertainty: feature vectors need to be estimated to predict future rewards, but can in principle be observed. In contrast, the group memberships need to be estimated because they are unobservable to begin with.
- The optimization over the selection vector, 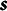 , is “sandwiched” between the two expected values discussed in the previous bullet point. It happens inside the expectation over the feature vectors, which are available at the time the selection is made, but outside the expectation over the unknown group memberships.
- There always exists a deterministic optimal selection policy. This follows directly from the fact that in (A.13) a function that is linear in the optimization variable, 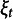 , is maximized over a probability simplex. Therefore, at least one vertex attains the maximum, and any vertex of a probability simplex corresponds to a single point mass on the respective outcome.
- The update of the posterior distribution detailed in Section 3.1 depends on the selection, but not on the *selection policy*. This is the case since the selected test takers only enter via the marginalization in (24). The latter requires knowledge of the selection, 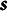 , but is independent of how this selection was made.
- In principle, it is not necessary to track 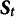 and 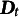 separately. Both can be combined into a single vector that indicates if the respective test taker is known to be a member of the critical group, of the reference group, or if their group membership is unknown. We decided to go with the slightly lengthier notation since we believe it to be conceptually simpler.

This concludes the discussion of the optimal selection policy. Based on the insights gained, we now turn to the design of approximate selection policies that are practical to implement.

## 4 Approximate methods

For the reasons discussed above, implementing the optimal selection policy is prohibitively complex, even for small *N* and *T*. In this section, we present heuristic policies that are significantly simpler yet sufficiently flexible and powerful to be useful in practice.

As discussed in Section 3.2, the update of the posterior distribution is independent of the selection policy. Therefore, we treat the two separately: first, we propose a variational approximation of the posterior distribution, and, second, we discuss three selection policies that can be used in conjunction with the approximate posterior update.

### 4.1 Approximate posterior update

The approximation method proposed in this section is essentially an expectation maximization (EM) algorithm (Dempster et al., 2018). However, it also incorporates ideas from the mixture-based extended auto-regressive model, see (Šmídl & Quinn, 2006, Chapter 8), and basic copula theory (Jaworski et al., 2010). First, each element of the feature vector, 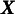 , is assumed to follow a distribution from the exponential family. That is,
(26)

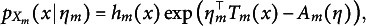

with natural parameters 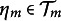 , base measure 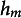 , sufficient statistic 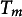 , log-partition 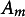 and 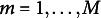 . Correlations between the elements of 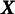 are modeled via a Gaussian copula. To this end, we define the random vector 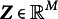 as
(27)

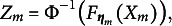

where 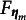 denotes the cumulative distribution function (CDF) of 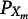 , and 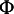 denotes the CDF of the standard normal distribution. The random vector 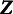 is then modeled as normally distributed with mean zero and covariance 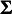 , that is, 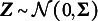 . It can be shown that (26) and (27) define a class of distributions with PDFs
(28)

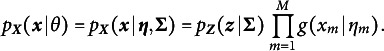

Here, 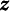 is calculated from 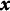 according to (27), 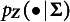 denotes the PDF of a zero-mean multivariate normal distribution with covariance matrix 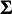 , and we defined
(29)

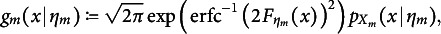

where 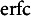 denotes the complementary error function.

The model in (28) is assumed to hold under both hypotheses, that is, for test takers in the critical group and in the reference group. The corresponding parameters and functions are denoted by an additional subscript 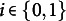 , for example, 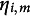 . Note that the exponential families of distributions do not need to be of the same type under both hypotheses.

A priori, we assume the free parameters to be independent and distributed according to
(30)

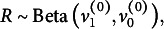



(31)

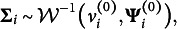



(32)

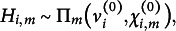

where Beta denotes the beta distribution, 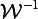 denotes the inverse Wishart distribution, and 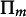 denotes the conjugate prior of the exponential family distribution corresponding to the density in (26). This distribution exists and its density is of the form
(33)

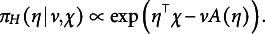

The parameters 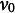 and 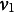 , which represent the effective number of observed samples under each hypothesis, are shared across all priors. Allowing for different initial effective sample sizes, 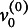 and 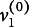 , is straightforward. We do not introduce this modification here, as it would significantly complicate the notation. In practice, however, it can be the case that, for example, one has more prior knowledge about the cheating rate distribution than the feature distribution.

While not guaranteed to accurately reflect the true prior knowledge in general, we consider the conjugate priors in (30)–(32) a useful and robust choice in practice—mainly for two reasons: First, as will become clear later in this section, conjugate priors significantly reduce computational complexity and eliminate the need for Monte Carlo sampling or numerical optimization in the inference step. Second, they are arguably a good fit for the problem at hand. Since the flagging procedure is assumed to run periodically, a natural prior for the parameters at time *t* is simply the posterior from the previous time instant, 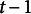 . In this chain, later priors are more accurate, as they incorporate more information. This effect is captured by the conjugate priors, which, by construction, remain of the same type after each administration, but increase their effective sample size, reflecting the larger set of training data. In the absence of training data, the *least informative prior* is obtained by setting the effective sample size to its minimum feasible value.

Based on the model specified above, we propose the following approximate posterior update. At time instant *t*, the approximate posterior distributions of *R*, 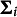 , and 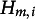 are given by
(34)

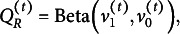



(35)

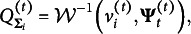



(36)

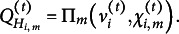

**Figure 2:**
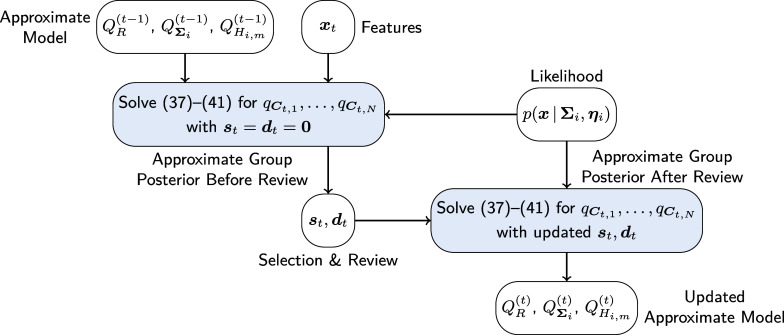
Graphical illustration of the approximate posterior update in (37)–(41).

The distribution parameters on the right-hand side, 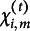 , 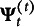 , and 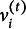 , are obtained from their previous values, 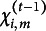 , 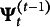 , and 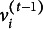 , by solving the following system of equations:
(37)

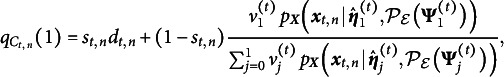



(38)

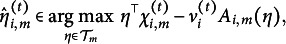



(39)

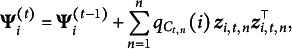



(40)

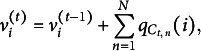



(41)

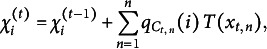

where 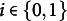 and 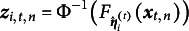 . The proposed approximate posterior update for a given selection policy is summarized in Algorithm 1, a graphical illustration in analogy to Figure 1 is shown in Figure 2.

Note that the posterior update in Algorithm 1 is run twice—once before the test takers are selected and once after the reviews are completed. The purpose of the first update is to obtain the approximate posterior distributions of the group indicator variables, 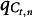 . The selection policies discussed in the next section are all based on these distributions. The purpose of the second update is to refine the estimates of 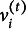 , 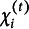 , and 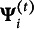 by incorporating the outcomes of the reviews. The difference between the first and second runs is reflected in (37), where the probability that the *n*th test taker of the *t*th administration is a member of the critical group is estimated from the observed features in case the test taker has not been reviewed ( 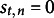 ), or is set to the true label, 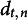 , if the review outcome is known ( 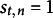 ).

**Figure:**
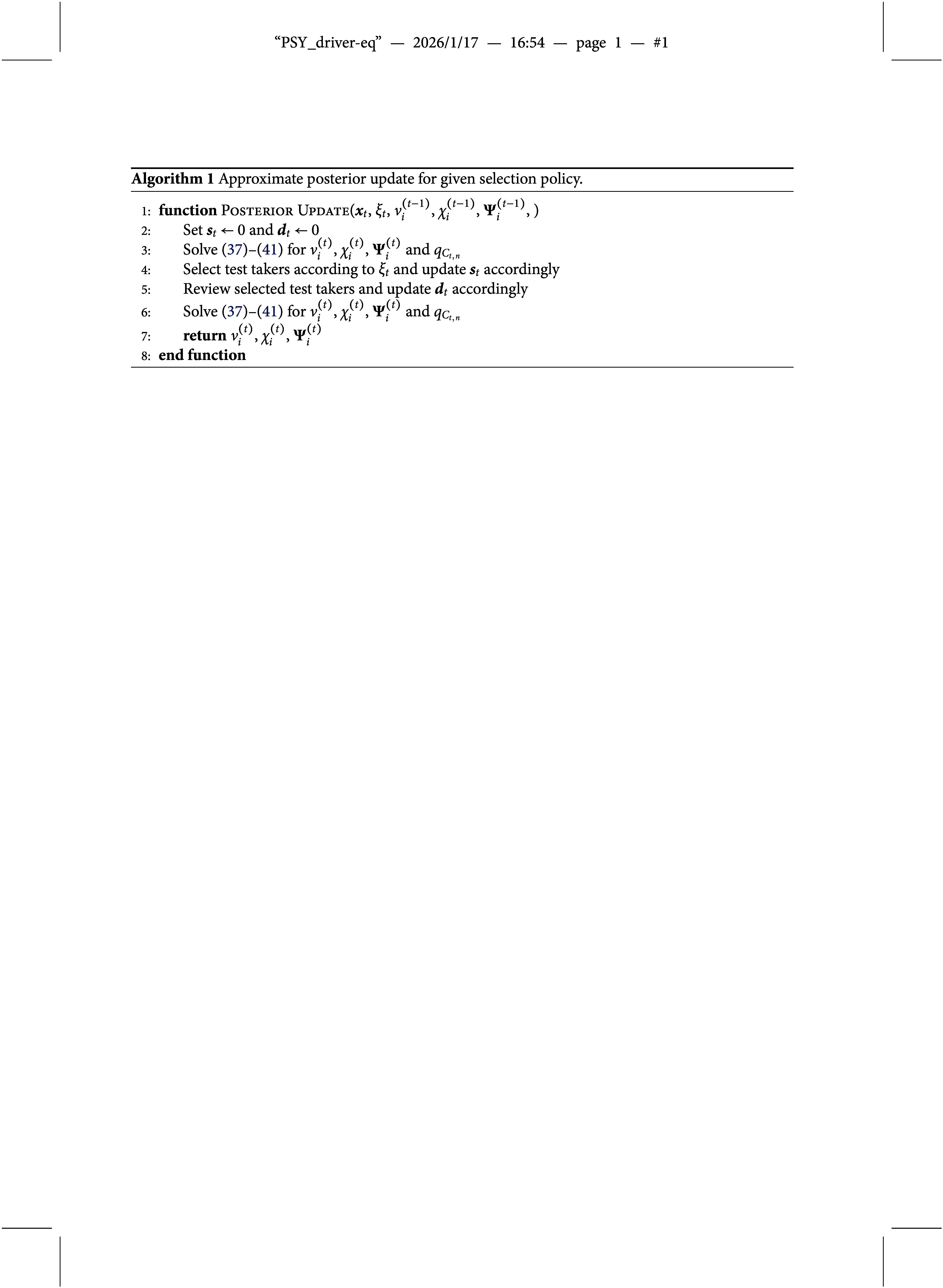

The idea underlying the update equations in (37)–(41) is to start with the priors in (30)–(32) and approximate the posterior distributions via a mean-field variational approximation (Blei et al., 2017; Šmídl & Quinn, 2006). By construction, this approximation yields posterior distributions that are of the same type as the priors, and whose parameters are determined implicitly by a system of equations. However, this system is still hard to solve since it involves expectations taken with respect to the current posterior distribution. In order to simplify the evaluation of the latter, we use maximum a posteriori probability (MAP) estimates as an approximation of the expected values. More precisely, to evaluate the posterior group membership probabilities in (37), we calculate MAP estimates of the marginal parameters in (38). Once the marginal parameters are fixed, the observations, 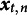 , can be mapped to the “Gaussian domain,” 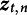 , via (27). The latter can then be used to update the covariance posterior in (39). The effective sample sizes and the parameters of the posterior distributions of the marginal parameters are updated according to (40) and (41). Of course, since all updates are coupled, they cannot simply be performed in sequence, but need to be solved jointly. We will further elaborate on this aspect later in this section.

Finally, note that in the model updates (39)–(41), the posterior group probabilities are used as weights, that is, the observation 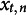 contributes to the model of the critical group with weight 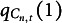 and to the model of the reference group with weight 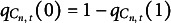 . This observation will later be used in the derivation of heuristic selection policies.

Before concluding this section, we briefly discuss some implementation aspects of Algorithm 1:

- There are various ways of solving the equation system in (37)–(41) in practice. A natural approach is to iterate over the updates until all parameters and probabilities have converged. Alternatively, one can use standard numerical solvers. In this case, it is useful to consider only 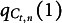 , 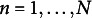 , as free variables since all other variables are fixed once all 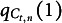 are given. Note that this means that the equation system is of size *N* in the first update step of Algorithm 1 and of size 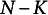 in the second.
- Depending on the choice of the marginal distributions, the optimization problems in (38) may not have a closed-form solution. However, even if (38) needs to be solved numerically, the underlying distributions are univariate so the number of parameters is typically small. Moreover, for given 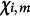 and 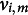 , the 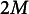 problems in (38) can be solved in parallel.
- In (37), the covariance matrix of the feature distribution is projected on the elliptope of correlation matrices. This projection is not strictly necessary. However, in our experiments, it leads to an improved performance. This is likely the case because the true 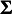 has unit diagonal elements if the model in (26) holds exactly. However, in practice, replacing the projection in (37) with the maximum likelihood estimate, 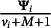 might improve the performance in some cases.
- In practice, 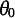 , 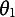 , and *r* do not necessarily remain constant for all 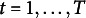 . While initially the Bayesian model can and will adapt to variations, the more samples are observed, the higher its “inertia” becomes. In order to counteract this effect, past observations can be down-weighted in the posterior update. For example, an exponential decay can be introduced by making the substitutions 
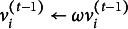
 , 
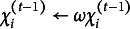
 , and 
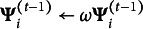
 in (39)–(41), where 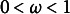 is a weight that balances adaptivity and steady-state performance—compare (Šmídl & Quinn, 2006, Section 8.6).

### 4.2 Approximate selection policies

The algorithm proposed in the previous section offers a mechanism to track the posterior distributions of the model parameters for a given selection policy. However, it does not answer the question of how to choose a selection policy in the first place. This question will be addressed in this section.

We propose three selection policies: A detection-greedy policy, an information-greedy policy, and a mixed policy that balances the information-greedy and detection-greedy approaches depending on the estimated accuracy of the current model.

#### 4.2.1 Detection-greedy selection policy

Consider the optimal selection policy defined by the optimization problem (9). Adopting a greedy approach and ignoring the expected future rewards, this problem simplifies to
(42)

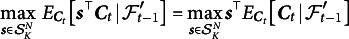



(43)

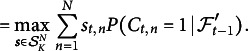

That is, the optimal greedy selection policy is to select the *K* test takers with the highest posterior probability of being members of the critical group.

Although the detection-greedy policy is much simpler than the optimal one, evaluating 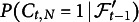 will still be too complex in most practical cases. Therefore, the heuristic policy suggested here is to select test takers based on the approximate probability 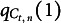 . This leads to the following selection policy:

Selection Policy 1 **(Detection-Greedy).**

At time instant *t*, the detection-greedy selection policy, 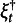 , is defined as
(44)

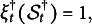

where
(45)

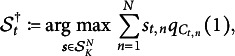

with 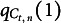 defined in (38). In words, 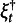 flags the *K* test takers that are most likely to be members of the critical group according to the approximate posterior probability 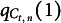 .

The detection-greedy policy is a natural choice, and, as the numerical examples in Section 6 will demonstrate, works well in many cases. However, by design, it does not take into account the benefits of potential model improvements that could be achieved by reviewing particularly informative cases. The second policy aims to identify the latter.

#### 4.2.2 Information-greedy selection policy

The idea underlying this selection policy is not to review the cases that are most likely to lead to detections, but to review the cases that provide the *most information*. In terms of the optimal selection policy in (9), this roughly corresponds to maximizing only the expected reward term, 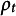 , and ignoring the immediate reward 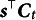 . In this sense, the information-greedy policy complements the detection-greedy policy. However, as was the case for the latter, directly maximizing 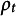 is computationally infeasible. We propose the following heuristic:

Selection Policy 2 **(Information-Greedy).**

At time instant *t*, the information-greedy selection policy, 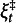 , is defined as
(46)

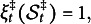

where
(47)

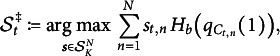




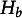
 denotes the binary entropy function, and 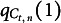 is defined in (37). In words, 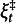 flags the *K* test takers whose approximate posterior group-membership distribution admits the highest entropy.

The rationale for the information-greedy selection policy is that resolving cases with the highest ambiguity has the greatest effect on model updates. More specifically, in (39)–(41), observed samples contribute to the parameter updates of the posterior distributions with weights 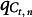 . Reviewing cases that are highly likely to belong to a certain group has little effect on these weights. For example, reviewing a case with 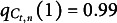 will most likely result in a detection, changing the weight from 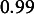 to 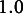 . This small change has a negligible impact on the model. In contrast, reviewing cases with high entropy, that is, 
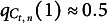
 , has a strong impact: Without review, the sample contributes equally to both models, reducing separability. With review, it contributes exclusively to the correct model, increasing separability.

On its own, the information-greedy policy is of limited use, as it does not explicitly aim to maximize the number of detections.^2^ However, it serves as a useful building block for an adaptive policy that balances model fit and detection rate (DR). Such a policy is proposed next.

#### 4.2.3 Mixed selection policy

The detection-greedy selection policy is a good choice when the learned model is close enough to the true one to produce sufficiently reliable predictions. However, the information-greedy selection policy might be useful to reduce the time and samples needed to reach this point. The idea underlying the proposed mixed policy is to track the model fit and balance the two policies accordingly. However, quantifying model fit is not straightforward. As discussed before, the optimal measure in (9) is too complex to evaluate in practice. Possible proxy measures include, for example, the variance or entropy of the posterior distribution. However, translating the latter into a useful exploration/exploitation trade-off is a non-trivial problem itself.

The heuristic proposed here is to use the detector calibration as a measure of the model accuracy. The underlying idea is the following: If the learned model is a good fit, the resulting (approximate) posterior probabilities will be reasonably accurate. That is, assuming that the posterior probabilities of the *K* selected test takers being critical group members are 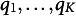 , the number of critical group members detected in the review process should be close to the sum of these probabilities. More precisely, it should hold that
(48)

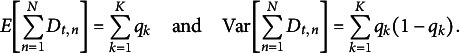

If the number of true positives is significantly lower than one would expect according to (48), the model likely needs improvement. This motivates the following selection policy:

Selection Policy 3 **(Mixed).**

Let 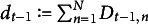 be the number of detected critical group members in the administration at time instant 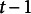 , and let
(49)

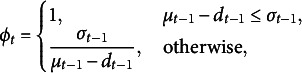

where 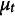 and 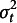 denote mean and variance defined in (48). In the administration at time instant *t*, first select 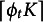 test takers according to Selection Policy 1, then select the remaining 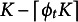 test takers according to Selection Policy 2, where 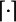 denotes the ceiling operation.

In words, 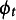 is the reciprocal of the number of standard deviations 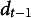 falls short of its mean. Note that, as long as 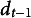 remains within one standard deviation, the mixed policy reduces to the detection-greedy policy.

Instead of fixing the shares of test takers selected according to each policy, one can also use a randomized strategy, where test takers are selected sequentially and selection policies 1 and 2 are used with probabilities 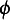 and 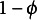 , respectively.

Finally, note that the distribution of 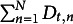 is known, namely, it is a Poisson binomial distribution with parameters 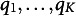 (Tang & Tang, 2023). Therefore, the probability of observing a certain number of critical group members can be calculated exactly, and can be used to refine Selection Policy 3. However, given the heuristic nature of the proposed selection policies, such refinements are unlikely to be worth the extra computational cost.

## 5 Performance bounds

Before investigating the performance of the proposed methods numerically, it is useful to establish theoretical bounds. In this section, two such bounds are presented. The first bound is obtained by assuming the model ( 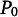 , 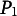 , and *r*) to be known. In this case, the problem in (8) can be solved exactly. The second result provides a simple, distribution-independent bound on the DR of any selection policy in terms of *N*, *K*, and *r*.

Assuming 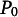 , 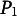 , and *r* to be known eliminates the need to learn the model and, in turn, decouples different test administrations. Therefore, the bounds in this section are given for a single administration, and the respective subscripts are omitted.

For the first bound, we establish a connection between the error probabilities of an optimal test for 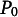 against 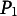 and the objective function in (8).

Lemma 1. For any 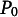 , 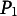 , and *r*, it holds that
(50)

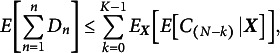

where 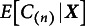 denotes the *n*th-order statistic of 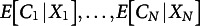 . It holds that
(51)

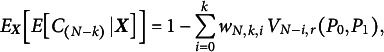

where
(52)

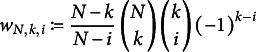

and
(53)

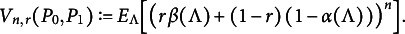

Here, 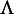 is a random variable that follows a shifted hyperbolic secant distribution, with PDF
(54)

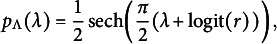

and 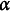 and 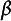 are the error probabilities of a likelihood ratio test for 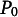 against 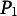 :
(55)

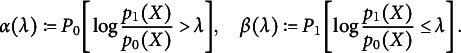

The lemma is proven in Appendix B. It is of interest since it provides a way of decomposing the cost function on the left-hand side of (50). The feature distributions, 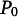 and 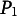 , and the share of critical group members, *r*, only enter (51) via 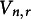 . Assuming that sufficiently many terms 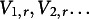 have been calculated, the effect of changes in *N* and *K* can be investigated by merely changing the weights 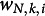 , which can be calculated efficiently for realistic administration sizes. If the effects of changing 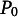 and 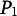 are of interest, evaluating the bound in (50) is computationally expensive since all 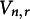 have to be recalculated or simulated from scratch. In such cases, simply simulating the selection procedure under the distributions of interest is likely to be easier and faster.

The second result in this section is a simple bound on the expected detection *rate* of any selection policy. To derive it, we assume that no detection errors occur or, equivalently, that the selection policy has direct access to 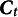 . In this case, critical group members are missed only if their number in the administration exceeds *K*. This leads to the following bound:
(56)

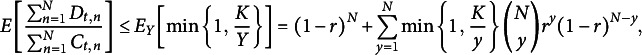

where 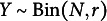 , with 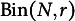 being the binomial distribution with *N* trials and success probability *r*. Note that, in (56), we define the DR to be one if the number of critical group members is zero (“no missed detections”).

The bound in (56) is somewhat trivial, but it can be useful to quickly determine lower bounds on *K* or acceptable ranges for *r*. For example, assuming that 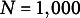 and 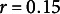 , if one seeks to detect at least 90% of critical group members, 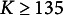 has to hold irrespective of the feature distributions and the selection policy.

## 6 Numerical examples

In this section, we present two numerical examples to illustrate the results discussed in the previous sections. The first example uses synthetic data to evaluate the proposed heuristics in a controlled setting. The second uses real-world data, which will be described in more detail below. For context, we compare the proposed procedure to three variants of a reference procedure that combines partial labeling with off-the-shelf classifiers.

In both examples, the marginal feature distributions in (26) are assumed to be beta distributions, that is,
(57)

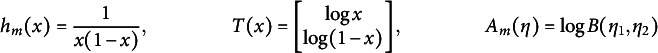

for all 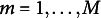 , where *B* denotes the beta function. Every 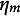 is assumed to have the same prior, namely,
(58)

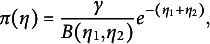

where 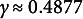 . The prior of 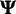 is assumed to be 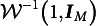 , and the prior of *R* is assumed to be 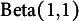 . These choices correspond to initial parameters 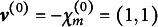 and 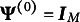 and are least informative in the sense discussed in Section 4.1.

To clarify, the proposed method is by no means limited to beta marginals. The main reason for using them here is that in the application that motivated this work, plagiarism detection in a large language test, the feature vectors consist of various text similarity metrics ranging from zero (no similarity) to one (identical texts). These similarity metrics were empirically found to be approximately beta distributed. Beyond this particular application, beta distributions are often a natural choice when the features themselves can be interpreted as probabilities. This is the case, for example, when fusing the output of multiple classifiers.

In both examples, we used the same priors for test takers in the critical and reference groups. That is, no prior knowledge about the feature distributions was assumed. This choice was made for two reasons: First, it corresponds to a worst-case scenario of no prior knowledge and in turn yields conservative performance estimates. Second, our goal was to evaluate how well the proposed method learns from data and leverages review outcomes. This is most apparent when the model is learned from scratch. In practice, of course, prior information can and should be incorporated into the model.

Python code to replicate the experiments in this section is available at Fauss (2025).

### 6.1 Reference procedure

As discussed in Section 1, the sequential semi-supervised flagging problem addressed in this article is non-standard, and none of the methods found in the literature met all our requirements. Thus, there is no obvious reference procedure to compare the proposed one to.

In order to at least establish a performance baseline, we propose combining off-the-shelf classifiers with *pseudo labeling* (PL), a generic method for learning from partially labeled data. PL was proposed by Lee (2013) and is still widely used today (Kage et al., 2025; Zhai et al., 2019). Its idea is to use a model trained on labeled data to assign labels to unlabeled data. The predicted labels, called pseudo-labels, are then treated as if they were true and used to train the model alongside the original, labeled samples. To reduce noise, pseudo-labeling is usually applied selectively, for example, by only assigning pseudo-labels to samples that were classified with high confidence.

In principle, PL can be combined with any classifier. However, given the complications of the flagging problem, we consider classifiers with the following properties:

1. The classifier outputs *soft labels* (probabilities or scores) that allow samples to be ordered from least to most likely to belong to the critical group.
2. The classifier can be trained via *stochastic gradient descent* (SGD) (or a variant thereof), which naturally lends itself to learning from sequential data.
3. The classifier can operate in a *small-to-medium sample size* regime, minimizing the need for pretraining before use in operation.

**Figure:**
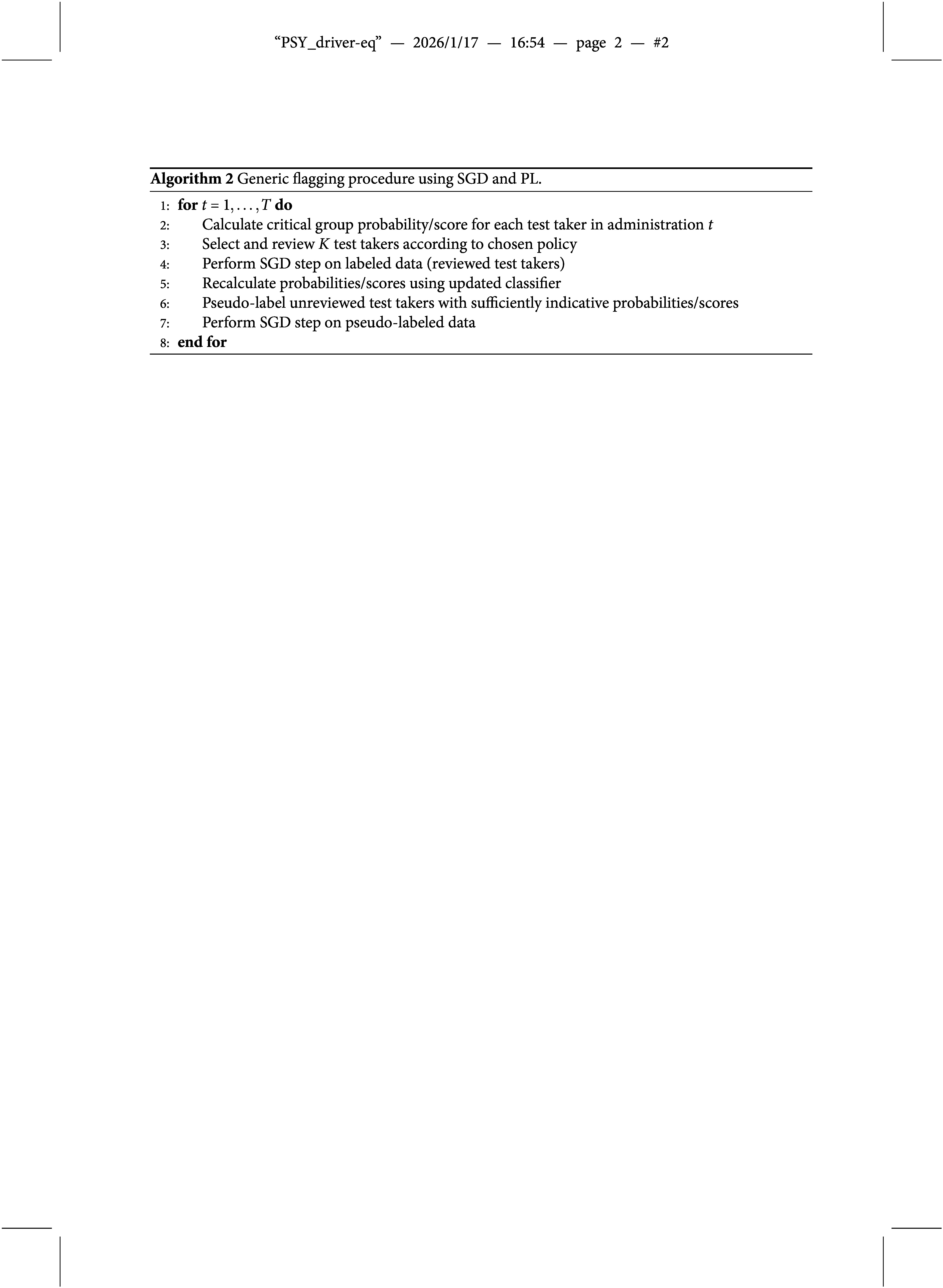

Note that some advanced methods that are routinely used in more standard settings do not meet the requirements stated above. For example, deep neural networks typically require large datasets to reach their full potential (Sun et al., 2017); many ensemble methods, such as random forests and boosting, are inherently designed for static datasets and not well-suited for sequential learning without substantial modification (Dietterich, 2000; Saffari et al., 2009); and methods such as *k*-nearest-neighbors or decision-tree classifiers require additional, often sample-hungry calibration procedures to convert hard labels to soft labels (Niculescu-Mizil & Caruana, 2005; Zadrozny & Elkan, 2002).

For the experiments in this section, we used three different classifiers: logistic regression, a linear support vector machine, and a fully-connected neural network with a single hidden layer and rectified linear unit (ReLU) activation function. These classifiers are well-established, highly popular among practitioners, and efficient implementations are readily available in various programming languages. The resulting procedures are referred to as PL-LR, PL-SV, and PL-NN, respectively. The underlying algorithm for combining PL with SGD-based classifiers is summarized in Algorithm 2.

All three reference procedures were implemented via the scikit-learn framework (Pedregosa et al., 2011). PL was implemented as follows: During the first 10 administrations, only reviewed samples were used for training. After this warm-up period, the threshold for PL positive samples (critical group members) was set to the lowest predicted probability or score among the confirmed positive samples in the current administration. For example, if three cases were identified as critical group members during the review, and the classifier had predicted these outcomes with probabilities 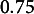 , 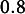 , and 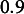 , then all samples whose predicted probability of belonging to the critical group exceeds 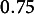 were assigned a positive pseudo-label. Negative pseudo-labels (reference group members) were assigned analogously.

The reference procedures were combined with a detection-greedy flagging policy, that is, the *K* samples with the highest likelihood of belonging to the critical group were selected for review. We chose this policy because it can be implemented irrespective of whether a classifier outputs probabilities or scores and, as will be shown shortly, the proposed Bayesian procedure typically performed best with a detection-greedy policy.

In addition to the PL and selection rules, all three classifiers have hyper-parameters that need to be set. However, in the sequential settings of the flagging problem, hyper-parameter optimization is non-trivial and requires custom methods (Gama et al., 2014) or substantial modifications of methods for static datasets (Shumway & Stoffer, 2022). Since these complications are well beyond the scope of this article, we decided to set the hyper-parameters by directly optimizing the observed performance. This is clearly not possible in practice, but provides useful insights into the *potential* performance of different methods.

Finally, we focused on tuning two important hyper-parameters: the learning rate used in the SGD steps, which is critical for balancing fast learning with good steady-state performance, and the width of the hidden layer of the neural network. The remaining hyper-parameters were left at their default values in scikit-learn (version 1.6.1). The learning rate was optimized via a grid-search over the interval 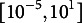 with 61 logarithmically-spaced grid-points. The width of the hidden network layer was optimized via a grid search over the interval 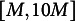 in steps of 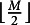 , where 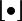 denotes the flooring operation. Results are reported for the parameter values that, averaged over 20 runs, showed the best performance. More details will be discussed in the course of this section.

While clearly not exhaustive, we believe that the three PL procedures detailed above provide a useful baseline for how well the flagging problem can be solved by combining off-the-shelf components.

### 6.2 Synthetic data

For the first example, we consider 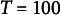 test administrations with 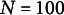 test takers each, approximately 20% of which are members of the critical group ( 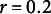 ). The selection is made based on 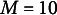 features that admit beta-distributed marginals with the following, randomly generated parameters:
(59)

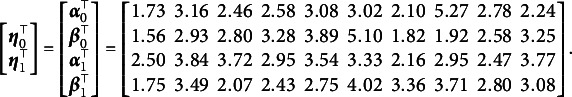

For members of the reference group, the features were assumed to be uncorrelated, 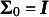 . For members of the critical group, the correlations were modeled as 
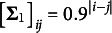
 .

**Figure 3:**
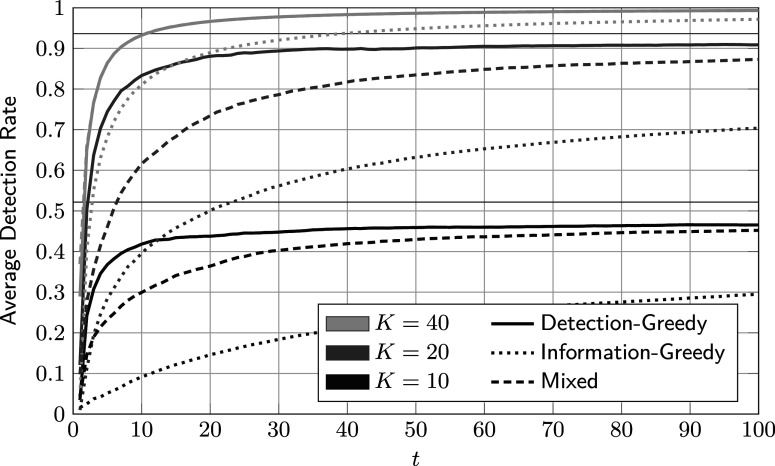
Average accumulated detection rate of the proposed policies against the number of administrations for different review sizes *K*. *Note*: Here, 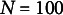 , 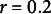 , and the parameters of the feature distributions are given in (59). The thin horizontal lines indicate the upper bound on the detection rate in (56).

Figure 3 shows the average DR of the selection procedure as a function of the number of administrations. Note that DR here refers to the accumulated fraction of detected members of the critical group, that is,
(60)

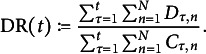

The results were averaged over 20 runs, and identical feature distributions were used for every run. By inspection of Figure 3, it can be seen that the detection-greedy policy performs best in this example, irrespective of the number of reviewed test takers. The DR of the mixed policy, which balances detection and model improvement, first grows significantly more slowly than that of the detection-greedy policy, but ultimately converges to approximately the same value. This means that the mixed policy first focuses on learning the model, at the expense of a lower DR. However, once the model is sufficiently accurate, it performs just as well or even better than the detection-greedy policy. In contrast, the information-greedy policy shows significantly lower DRs across all experiments. This is expected, as it is not designed to maximize the DR. In fact, it might be somewhat unexpected that the DR does not converge to 50%. In this example, this is the case because the entropy of the posterior group distribution tends to be higher for members of the critical group than for members of the reference group. As a consequence, the information-greedy policy flags more test takers from the former. In general, we observed that the information-greedy policy tends to flag more members of the *smaller* group, which is the critical group in this case.

From Figure 3, one may draw the conclusion that the mixed selection policy is redundant, given that it performs worse than the simpler detection-greedy policy. However, this result does not convey the whole picture. First, for both 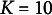 and 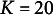 , the mixed policy only performs worse than the detection-greedy policy in the early test administrations, with the largest gap between the two occurring at around 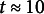 . As *t* increases, the gap between the two curves narrows, meaning that the mixed policy performs equally well or even better for virtually all later administrations. Consequently, the mixed policy can be preferable in cases where the first administrations are less critical and can be used primarily for data collection. For example, new items are commonly piloted before being used in high-stakes tests (von Davier, 2010).

**Figure 4:**
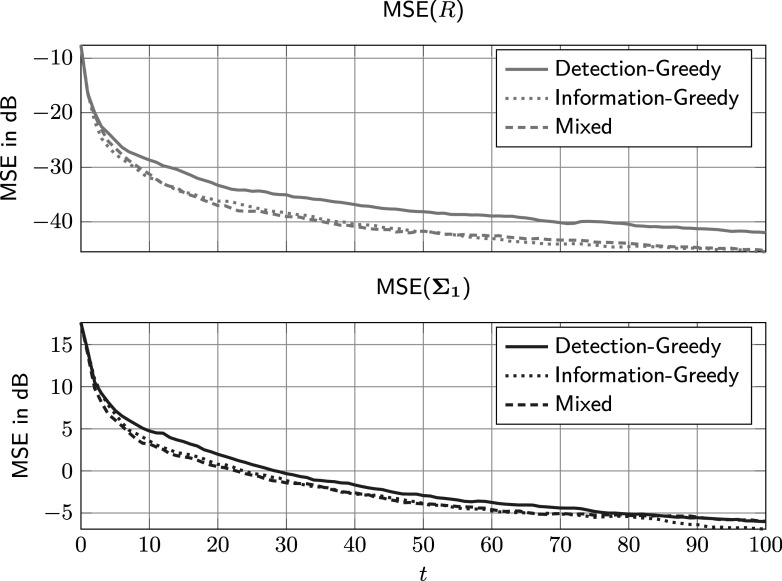
Average MSE against the number of administrations. *Note*: Here, 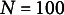 , 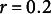 , 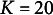 , and the parameters of the feature distributions are given in (59).

A second advantage of the mixed policy is that it leads to a more accurate model. Figure 4 shows plots of the average mean squared error (MSE) of two parameters, *R* and 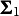 , over the number of administrations. The MSE was calculated with respect to the posterior distribution at the time and again averaged over 20 runs. For all three policies, the large initial model errors steadily decrease, meaning the procedure does indeed learn the model from the data. However, the information-greedy and the mixed policy learn the parameters faster. Consider, for example, the MSE of *R* in the top plot of Figure 4. While it takes the detection-greedy policy approximately 75 administrations to reach 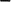 40dB, the information-greedy and mixed policy only need half as many samples to reach the same error level. For the error in the covariance matrix estimate, shown in the lower plot of Figure 4, this difference is less pronounced, but still noticeable. For example, the information-greedy and mixed policy reach the 0dB level approximately 10 administrations or 1000 samples earlier than the detection-greedy policy.

**Figure 5:**
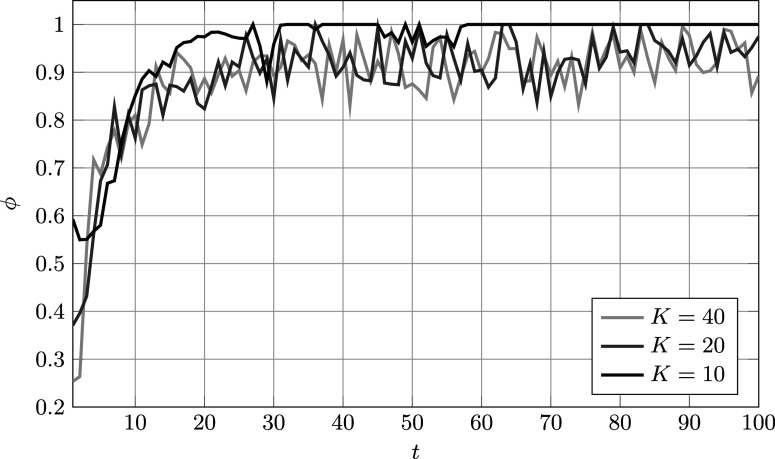
Average values of 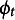 in (49) against the number of administrations. *Note*: Here, 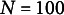 , 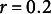 , 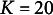 , and the parameters of the feature distributions are given in (59).

Finally, some insight into the mixed selection policy can be gained by observing 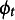 , defined in (49). Its value, averaged over 20 runs, is plotted against the number of administrations in Figure 5. It can be seen that initially a substantial share of samples is selected according to the information-greedy policy, which is in line with the quicker model fit/learning discussed above. However, after approximately 30 administrations, 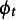 reaches a steady state in which it fluctuates around approximately 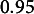 , meaning that most reviewed samples are selected according to the detection-greedy policy. Interestingly, the observed 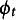 curves are almost identical for 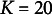 and 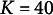 . Only for 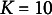 the behavior is slightly different, with 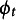 being strictly one for most administrations. This is likely a consequence of the second-order moment approximation used to calculate 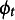 , which becomes more accurate as *K* increases. Nevertheless, the observed quick convergence to a purely detection-greedy policy is beneficial in this case since for 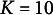 the procedure operates in a regime where the number of reviews, not the model fit, constitutes the main bottleneck.

The results discussed so far illustrate the trade-off between DR and model accuracy. On the one hand, the model needs to be “good enough” to reliably detect critical group members. On the other hand, investing too many samples into model improvements can lead to situations in which an unreasonable number of administrations is required to compensate for the lower initial DR. Based on our experiments, we conjecture that, as a rule of thumb, in cases where a high DR is the main or only goal, the detection-greedy policy should be used. In cases where early administrations can be used for training, or when an accurate model is of concern, for example, to track error rates, feature correlations, or shares of critical group members, the mixed policy can be preferable.

**Figure 6:**
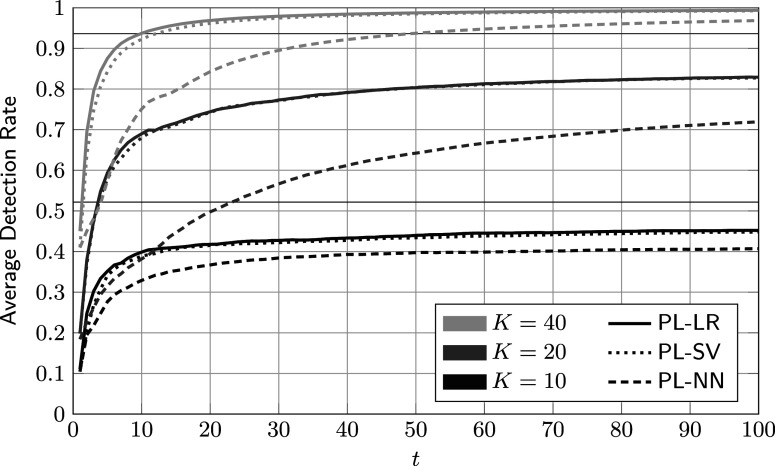
Average accumulated detection rates of reference procedures against the number of administrations for different review sizes *K*. *Note*: Here, 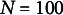 , 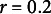 , and the parameters of the feature distributions are given in (59). The thin horizontal lines indicate the upper bound on the detection rate in (56).

To put the results shown so far into perspective, average DRs of the three reference procedures detailed in Section 6.1 are shown in Figure 6. The corresponding hyper-parameters are given in Table 1. By inspection, PL-SV and PL-LR achieve virtually identical performances for all values of *K*. PL-NN performs notably worse. We conjecture that this is the case because neural networks have significantly more free parameters and in turn require more training data. In this example, even the smallest neural network, with a hidden layer of width 30, has well over 300 free parameters. For comparison, the proposed Bayesian method has approximately 60 free parameters, and PL-LR and PL-SV both have fewer than 20.

**Table 1:** Hyper-parameters of reference procedures for different values of *K*

Procedure	Hyper-parameter	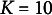	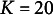	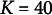
PL-LR	Learning rate	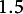	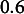	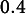
PL-SV	Learning rate	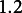	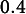	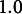
PL-NN	Learning rate	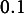	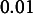	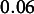
	Hidden layer width	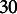	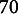	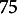

**Figure 7:**
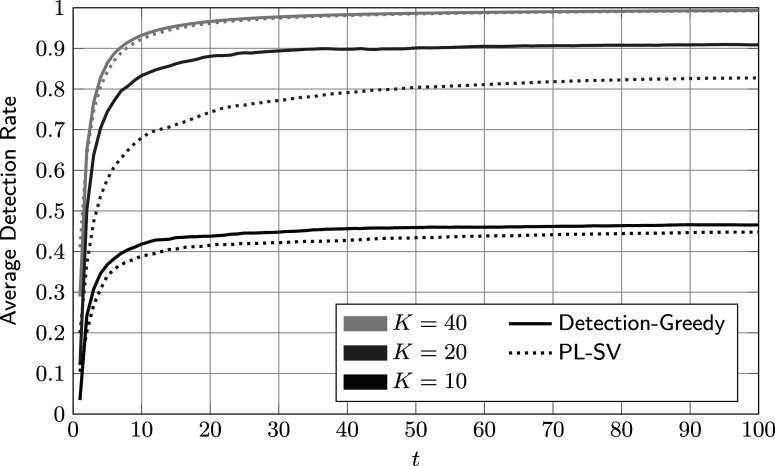
Comparison of average accumulated detection rates of the proposed detection-greedy policy and support-vector-based reference procedure. *Note*: Here, 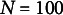 , 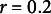 , and the parameters of the feature distributions are given in (59).

In Figure 7, we compare one of the best-performing baseline methods, PL-SV, to the detection-greedy variant of the proposed method. For large and small values of *K*, there are no significant performance differences. For 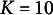 , both procedures appear limited mainly by the small number of reviews rather than their respective flagging or learning strategies. At the other end, for 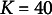 , there are enough labeled data points for both to quickly achieve virtually perfect separation. Only for 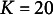 does the proposed method significantly outperform PL-SV. We conjecture that in this case, in which the share of reviewed cases equals the rate of critical group members ( 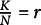 ), the proposed procedure’s more principled approach to data processing and model tracking pays off. Arguably, the case 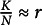 is also of practical relevance, as one would normally expect to review about as many cases as there are critical group members.

### 6.3 Real-world data

For the second example, we used real-world data collected from a large language assessment between November 2022 and March 2023. The dataset contains 2,595 data points, each a three-dimensional vector representing different similarity metrics between a submitted response and a potential source text it may have been copied from. Of the 2,595 responses, 745 ( 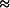 30%) were labeled as plagiarized (true positives) by human experts, and 1,850 ( 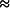 70%) as authentic (false positives), meaning the similarities did not constitute plagiarism under the program’s guidelines.^3^ The three similarity metrics used in this example all take values in the unit interval, with one corresponding to identical texts. For security reasons, we do not detail how the features were computed from the responses or which items were used for the study. For the purpose of illustrating the proposed flagging procedure, these specifics are of little importance. Interested readers may contact the authors directly.

An additional complication compared to the first example is introduced by the fact that, owing to pre-processing steps that will not be detailed here, the elements of the similarity vectors are left-truncated at 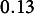 , 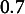 , and 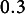 , respectively. In principle, the proposed algorithm can be adapted to use truncated exponential distributions. However, this extension is non-trivial and beyond the scope of this article. Instead, in this example, we disregarded the model mismatch and ran the flagging procedure with the same marginal distributions and parameters as in the first example. This approach is clearly suboptimal. However, we still consider the experiment meaningful since, in practice, model mismatches are inevitable and the flagging procedure should still perform acceptably well under such conditions.

We again used 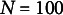 , 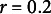 , and 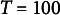 . The number of features, 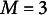 , was determined by the data. Samples from the critical and reference groups were generated via bootstrapping. That is, for each administration, we first sampled a vector 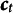 whose elements are independent Bernoulli random variables with success probability *r*. For each 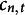 , the corresponding feature vector 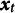 was then drawn uniformly and with replacement from the respective data set. In order to investigate the effect of the model mismatch, we fitted two multivariate truncated beta distributions to the true and false positives in our data set. These distributions were defined in analogy to those in Section 3.1, that is, the marginals are truncated beta distributions and the correlations are modeled via a Gaussian copula. This model largely eliminates the model mismatch and, in line with the assumptions underlying Lemma 1, was used to simulate the case in which the distributions 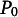 and 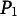 are known.

**Figure 8:**
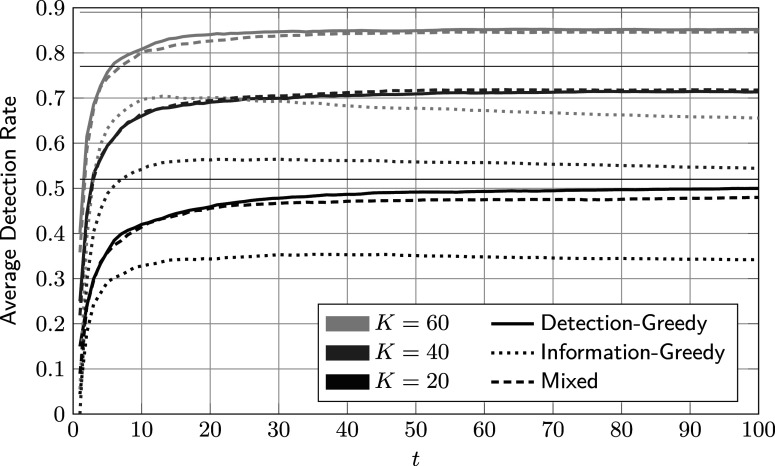
Average detection rate of the proposed policies against the number of administrations for different review sizes *K*. *Note*: Here, 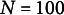 , 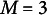 , and 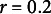 . The thin horizontal lines indicate upper bounds on the detection rate obtained by an “oracle” version of the proposed method that uses a fitted model from the beginning instead of learning it from the data.

In Figure 8, the average DRs of the three proposed policies are plotted against the number of administrations for different values of *K*. For comparison, we included bounds obtained by assuming 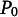 and 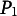 to be known, as explained above. In general, the DR curves behave similar to their counterparts in the first example, which corroborates the findings obtained from synthetic data. Interestingly, in the sample-rich regime ( 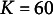 ), the performance of the information-greedy policy is on par with that of the detection-greedy and mixed policies. We conjecture that this is the case because samples from the smaller group tend to be more informative. For 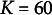 , the sample size is large enough for the policy to “saturate” the samples from the critical group.

**Figure 9:**
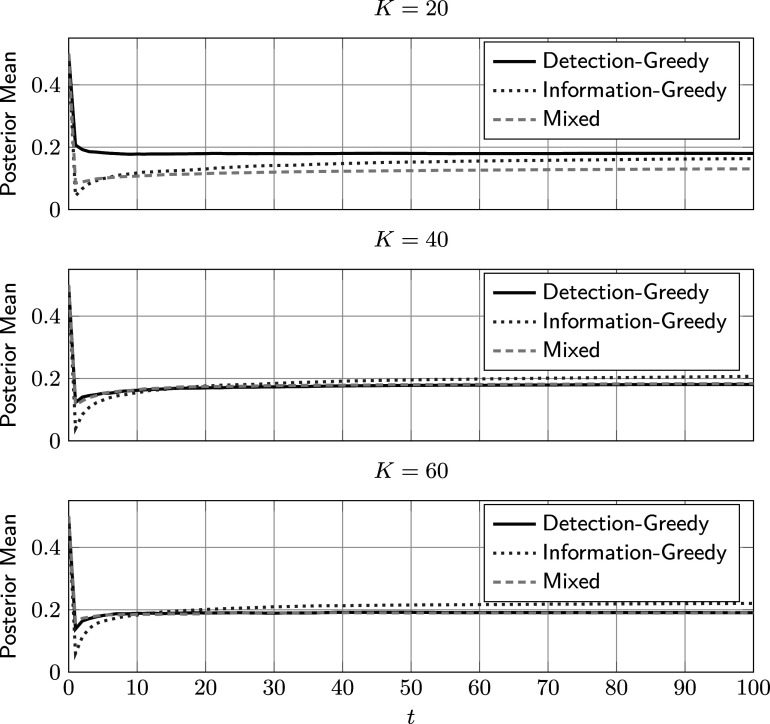
Average posterior mean of *R* against the number of administrations for different selection policies and values of *K*, with 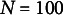 and 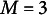 .

Next, we compare the model fit of the different policies. In lack of a ground truth for the other parameters, we look at the posterior mean of *R*. As can be seen in Figure 9, for all policies, the estimated rate of critical group members starts high, then quickly drops, and finally converges to a value close to 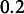 . This behavior can be explained as follows: Since *R* is assumed to have been drawn from a uniform prior, the initial mean is 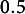 , which is significantly larger than the true value of 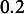 . As samples start coming in, the model adjusts to the lower observed rate of critical group members. However, for the first administrations, the uncertainty in the model is large, and the detection accuracy is still low. Therefore, only a fraction of the critical group members is detected, and the model goes from overestimating *r* to underestimating it. This overshoot effect is least pronounced for the detection-greedy policy, which has the highest DR in early administrations (compare Figure 8). We conjecture that this effect also contributes to the higher overall accuracy of the detection-greedy policy when 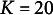 .

**Figure 10:**
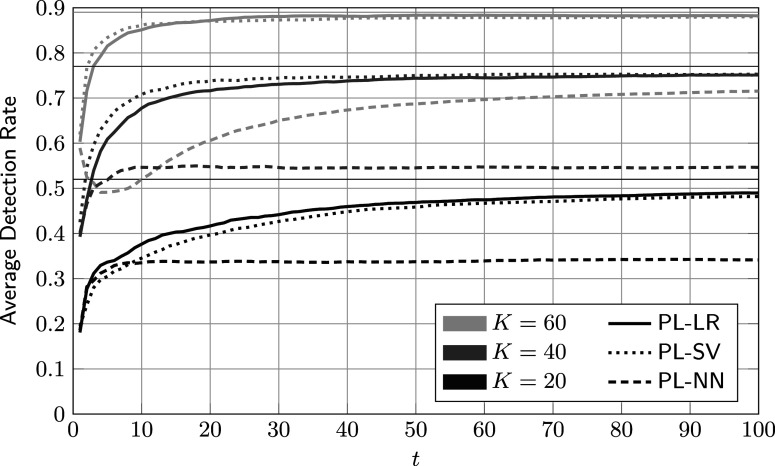
Average detection rate of reference procedures against the number of administrations for different review sizes *K*. *Note*: Here, 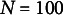 , 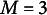 , and 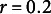 . The thin horizontal lines indicate upper bounds on the detection rate obtained by an “oracle” version of the proposed method that uses a fitted model from the beginning instead of learning it from the data.

The average DRs of the three reference procedures detailed in Section 6.1 are shown in Figure 10, and the corresponding hyper-parameters in Table 2. By inspection, PL-NN performs significantly worse than PL-SV and PL-LR. The latter admit virtually identical performance, with PL-LR performing slightly better for 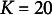 . This finding corroborates that neural networks do not seem to be a good fit for the sequential flagging problem: large networks require too many samples to learn the underlying model, and small networks lack the required expressive power. This problem could possibly be addressed by adaptively growing the network as more data become available, but exploring this approach is well beyond the scope of this section.

**Table 2:** Hyper-parameters of reference procedures for different values of *K*

Procedure	Hyper-parameter	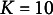	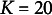	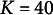
PL-LR	Learning rate	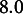	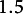	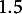
PL-SV	Learning rate	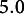	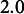	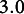
PL-NN	Learning rate	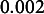	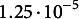	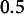
	Hidden layer width	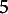	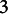	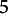

**Figure 11:**
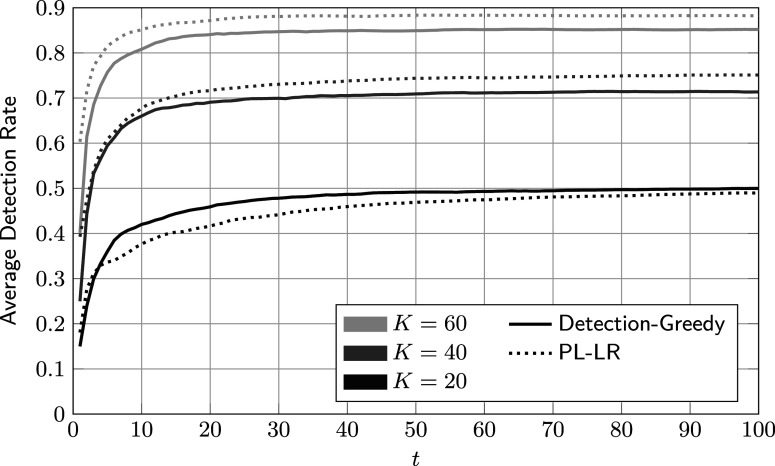
Comparison of average accumulated detection rates of the proposed detection-greedy policy and logistic-regression-based reference procedure. *Note*: Here, 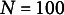 , 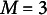 , and 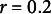 .

In Figure 11, we compare the proposed detection-greedy policy to the best-performing reference method, PL-LR. Interestingly, the latter slightly outperforms the former for larger values of *K*. We conjecture that this performance gap is mainly caused by the model mismatch discussed above. On the one hand, this result shows that, unsurprisingly, the proposed procedure is not necessarily the best choice under all circumstances. On the other hand, it is reassuring to see that even under moderate model mismatch, the proposed method admits DRs within three percentage points of the best reference method, and accurately estimates the share of critical group members among the test population (compare Figure 9). Also, note that PL-LR uses a close-to-optimal learning rate, which is unlikely to be the case in practice.

### 6.4 Summary

In summary, we conclude from the experiments in this section that the main strengths of the proposed procedure are as follows:

- Simplicity: The proposed procedure can be used without modifications for all sample sizes, time horizons, numbers of conducted reviews, feature dimensions, etc. In contrast, many alternative approaches, including the ones presented here, require the user to tune various hyper-parameters. For example, as can be seen from Tables 1 and 2, the optimal learning rates vary widely between different scenarios and can be far from common defaults, such as 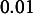 (TensorFlow Contributors, 2025) or 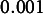 (PyTorch Contributors, 2025).
- Robustness: While not always being the best choice, the proposed procedure performed well in all of our experiments, even under moderate model mismatch. In combination with its simplicity, we believe that this makes it an excellent choice in practice.
- Fully Bayesian model: At any point in time, the user has access to the approximate posterior distributions of the quantities of interest and can perform additional inference as desired. While not always necessary, this allows for a much more systematic analysis of both the flagging decisions and the test taker population. Moreover, in particular when using the mixed policy, the procedure self-calibrates so that predicted probabilities can validly be interpreted as such.

As potential weaknesses, we identified the following issues:

- Model mismatch: While our experiments showed that the procedure can handle moderate model mismatch, it will break down under severe model mismatch. This means that the user should not apply the procedure blindly, but have some familiarity with the data of interest. For example, a simple sanity check is to verify that the assumed exponential family distributions roughly fit the true marginal distributions of the features.
- Compute: In our experiments, the proposed procedure typically took more time to run than the alternatives. This is partly due to its higher complexity and partly because our implementation is not as optimized as those provided by mature machine learning libraries. However, the time required to process a single administration averaged around 2s for 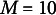 and 0.5s for 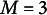 , making it real-time capable for most practical purposes.
- Numerical stability: The proposed procedure requires solving the equation system in (37)–(41). In our experiments, a simple fixed-point iteration converged most of the time. When it did not (fewer than 10 occasions across all experiments), the output of the last iteration was used. The effect of these inaccuracies appears negligible, but, in principle, one should be aware of potential numerical issues.

## 7 Conclusions and outlook

In this article, we have addressed the problem of automatically flagging test takers who display atypical responses or behaviors for further review by human experts. The primary goal has been to develop a selection policy that efficiently identifies individuals who require additional scrutiny, while also keeping the volume of reviews per test administration manageable. To achieve this, the flagging problem has been formulated as a semi-supervised learning task in a Bayesian framework. The corresponding optimal selection policy has been derived and discussed. Given the computational challenges in implementing this policy and updating the underlying posterior distributions, we have proposed a variational approximation along with three heuristic selection policies, each balancing exploration and exploitation in different ways. Through numerical experiments using both synthetic and real data, the performance of these approximate policies has been evaluated and compared with reference procedures based on off-the-shelf techniques.

Beyond the results presented in this article, there are variations and extensions of the proposed procedure that will be considered for future research. Some of these have already been mentioned in the text, but are included here for completeness.

- Assuming the number of reviews to be given and fixed has been convenient for the analysis in this article. In practice, however, other criteria can be more appropriate, such as the detection or false alarm rate. While such variations of the underlying problem formulation will lead to a conceptually similar procedure, we still expect that insights can be gained from explicitly deriving the corresponding selection policies.
- In some applications, it might not be appropriate to assume that the feature distributions and the share of critical group members remain constant between test administrations. For such cases, a variant of the proposed method is needed that tracks the changing parameters over time. Problems of this kind are known as (Bayesian) filtering and have been studied extensively in the literature (Särkkä & Svensson, 2023; Sayed, 2011).
- The flagging problem can be formulated under different assumptions about the available information. For example, the problem simplifies significantly if one replaces 
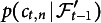
 with 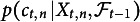 , that is, the decision whether to flag the *n*th test taker in the *t*th administration is restricted to depend only on the features extracted from the response under scrutiny. On the other hand, when tests are administered frequently, one may use data collected in multiple administrations, say *t*, 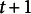 , and 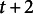 , to flag test takers in administration *t*, thus complicating the data fusion step.
- In this article, the population of test takers is assumed to be homogeneous, meaning that the feature distribution is identical for all test takers. This is not necessarily the case in practice, where different subgroups of test takers can admit different feature distributions. For example, in language tests, the feature distribution might be affected by a test taker’s first language. Similarly, in writing assessments, certain styles or structures can be common among subgroups of test takers that have the same educational background or used the same preparation material. Not taking such differences into account naturally leads to fairness concerns. A common approach to promote fairness is to add a constraint on an appropriate fairness metric. We expect this problem to be a non-trivial extension of the work in this article. For example, it significantly complicates the exploration/exploitation trade-off since, depending on the chosen fairness metric, various groupwise error probabilities need to be tracked and balanced. However, if implemented successfully, such a procedure would enable one to monitor fairness in real time, based on real-world data.

## Footnotes

1 An extension to a random number of test takers is possible, but will not be attempted in this article. However, the heuristics presented in Section 4 are applicable in both cases since they do not require *N* to be known in advance.

2 It will be shown later that high detection rates can arise as a side effect of information-greedy flagging.

3 Note that the  30% positive rate is based on a prefiltered sample and does not reflect operational rates.

## A Proof of Theorem 1

The theorem is proven inductively using techniques from sequential analysis and dynamic programming. Assume that for some  , it holds that

(A.1)

with  as defined in the theorem. We now show that if this is the case, then the optimal selection policy at time  is of the form (11). By Assumption 3, the selection policy  is  -measurable. It hence holds that

(A.2)

(A.3)

(A.4)

where the inner expectation is taken with respect to the distribution of

(A.5)

The probability mass function (PMF) of this distribution is given by

(A.6)

(A.7)

where the last equality follows from (4). The inner expectation on the right-hand side of (A.4) can hence be written as

(A.8)

Using (6), the inner most sum over  collapses to a single term

(A.9)

(A.10)

(A.11)

Substituting this result back into (A.8) yields

(A.12)

(A.13)

(A.14)

(A.15)

(A.16)

where the last equality holds by definition of  , and (A.15) holds since  defines a probability distribution over  . Note that equality in (A.15) holds if and only if  is of the form (11). To complete the induction step, we substitute (A.16) back into (A.4) and coarsen the  -algebra from  to  :

(A.17)

(A.18)

(A.19)

(A.20)

(A.21)

where the last equality holds by definition of  . Finally, the induction basis is given by  and  , for which (A.1) is trivially true.

## B Proof of Lemma 1

Once *N*, *K*, *r*,  , and  are given, the model no longer needs to be inferred from the data and the optimization problem in (8) reduces to a binary detection problem with known distributions. As a consequence, the cost function in (8) simplifies to

(B.1)

with  as defined in Lemma 1. The random variables  are independent and identically distributed and it holds that

(B.2)

(B.3)

(B.4)

Therefore, the CDF of  is given by

(B.5)

(B.6)

(B.7)

where  ,  and  and  are defined in (55). From (B.7), we can calculate the distributions of the order statistics  :

(B.8)

(B.9)

(B.10)

(B.11)

(B.12)

where (B.9) is shown in David & Nagaraja (2004, Chapter 2), (B.10) follows from the fact that

(B.13)

(B.11) is obtained by rearranging the lower-triangular sum over *j* and *i* (row-first vs. column-first), and (B.12) holds since

(B.14)

This identity can be shown by repeated application of Pascal’s formula (Weisstein, 2024) and has been verified by computer algebra systems.

Since  is a non-negative random variable, the expected values on the right-hand side of (B.1) can now be written as

(B.15)

(B.16)

(B.17)

In order to evaluate the integral in the last equation, we change the integration variable from  to

(B.18)

with the corresponding transformation of the infinitesimal

(B.19)

It hence holds that

(B.20)

(B.21)

(B.22)

where the second equality follows from (B.7), and  and  are as defined in the lemma. This completes the proof.
